# A Clinical Lecture on Typhoid Fever and Broncho-Pneumonia

**Published:** 1875-06

**Authors:** Francis Delafield


### A CLINICAL LECTURE ON TYPHOID FEVER AND BRONCHO-PNEUMONIA.

BY FRANCIS DELAFIELD, M. D.

*CASE I.*

GENTLEMEN :—I will ask your attention this morning to these diseased intestines and lungs. They were taken from the body of a young woman who died in this hospital. Her stay in the hospital was short, her previous history was obscure, and her symptoms were perplexing, so that I will not occupy your time with the consideration of the clinical aspect of the case.

First, then, let us examine the intestines. You will observe that in the lower part of the ileum there are a number of large and small ulcers, corresponding in their situation to the agminated and solitary glands. These ulcers have thick edges, their walls and floors look something like granulation tissue. In some of them we see yellowish sloughs of some size still adhering to the floors of the ulcers. If you turn over the intestines and look at their external surface, you will see in and beneath a peritoneal coat, corresponding to the position of the uleers, a number of small, rounded grayish bodies.

Such ulcers are one of the characteristic lesions of typhoid fever. In typhoid fever we find an enlargement of the agminated and solitary follicles of the ileum. This enlargment is followed by degenerative and ulcerative changes. In mild cases we find only small ulcers, involving perhaps only portions of the agminated follicles. Such have been the lesions in all the cases of typhoid fever which I have seen this fall.

In severe cases, however, the follicles attain a greater degree of enlargement, and the new growth of cells infiltrates the adjacent portions of the intestine. Instead of ulcerating subsequently, large portions of the enlarged follicles die and form sloughs of considerable size. This is the condition of affairs in the intestines of this patient.

Now, let us look at the lungs taken from the same patient.—They present us with a picture of acute broncho-pneumonia, such as we rarely see in the adult. Except for their size these lungs have exactly the appearance of the lungs of a child two years old with broncho-pneumonia. The bronchi are congested and lined with muco-pus. In the lung-tissue are numerous patches of hepatization, some red, some rather purple in color. In some of the lobules of red hepatization you will notice small grayish patches about the size of a pea. These correspond to small bronchi and zones of air-cells around them.

This condition of the lungs is one of the regular complications of typhoid fever. It is apt to be a very troublesome one. Not infrequently the lung lesions assume a chronic character, and the patient develops the symptoms of pulmonary phthisis.

These lungs may therefore serve to illustrate to you the form of broncho-pneumonia which may complicate typhoid fever, and may also serve as an example of the early stage of one of the varieties of pulmonary phthisis.

*CASE II.*

In this boy, gentlemen, you may observe the results of such changes in the lungs as you have just seen, if these changes persist and become chronic. The boy is fifteen years old. His father and mother died of phthisis. He has been badly fed, badly clothed, and constantly exposed to cold and wet. For about three months he has had a cough. This cough has increased in frequency and severity, and is now attended with profuse muco-purulent expectoration. He is now feeble, emaciated, and has hectic fever with night-sweats.

If we examine his chest we find evidences of bronchitis over the whole of the right lung, together with consolidation of its upper portion.

If we could look at this boy's lung at this moment we would find just such changes as you have seen in the lungs from the typhoid fever patient—but farther advanced. The bronchitis is no longer acute—but chronic. The bronchial mucous membrane is trabeculated and the fibrous coat of the bronchi is thickened. The hepatized lobules of lung tissue are no longer only red, but some are gray and some are cheesy. There is not only an inflammatory exudation filling the air-cells, but a new growth of fibro-cellular tissue between them.

What is the prognosis in this case, and what should be the treatment?

The *Prognosis* is not very bad. The right lung only is involved, and the process does not seem to have invaded a very large portion of that.

Of course, his recovery will depend very much upon the condition in which he is placed. In the hospital, in some respects he is well off, while in other respects he is unfavorably situated with regard to recovery.

With regard to treatment in these cases, it is always to be directed both towards the lesion of the lungs and towards the general condition of the patient.

The direct treatment of the lungs consists in making certain applications to the chest-walls. Of the various applications employed, I am very partial to dry cups, especially in the earliest stages. If we had seen this boy in the second or third week of his disease, we should have employed dry cups over the chest every day. Repeated cuppings, even now, will be very serviceable. These patients should not be cupped too hard. It is better, therefore, that you should apply the cups yourselves rather than rely upon professional nurses or cuppers. All you wish to do is to produce a free determination of the skin, without producing ecchymosis or making the skin sore. Used in this manner dry cups are useful, especially in the earlier stages; and even in the latter stages are of positive benefit. It has seemed to me sometimes in adults, in cases of broncho-pneumonia in which apparently the disease was about to go on into chronic phthisis, that the disease has been cut short by the steady, persistent application of dry cups. The success attending the use of this remedy depends very much upon the attention you yourself give to its application. If you do not use cups, some other form of counter-irritation should be employed, such as blisters, iodine, croton oil, etc.

The treatment of the patient's general health is to be regulated a good deal by the condition of the stomach and bowels. If the appetite is good and the bowels regular, cod-liver oil will be of service. Sometimes great benefit will be derived from the use of the syrup of the lacto-phospate of lime. This remedy is especially beneficial in treatment of children.

Churchhill's preparation of the hypophosphites is also valuable.

But these remedies are principally valuable in those cases in which the stomach and intestines are in good condition. If the stomach and intestines are not in good condition, the appetite poor and the bowels irregular, you must turn your attention towards these before crowding tonics, cod-liver oil, and food. For the purpose of improving the condition in this respect we may resort to the use of the remedies for the improvement of the appetite, such as the mineral acids, various preparations of iron, quinine, etc.; and sometimes these remdies can be used with very great benefit. Sometimes one article will do best, sometimes another.

If there is much vomiting, as not unfrequently there is, you will find that relief can be obtained sometimes by the use of a mineral acid and sometimes by the use of an alkali. Sometimes dilute hydrocyanic acid will be of benefit. With regard to cough, if it is not troublesome let it remain untreated. If sufficiently severe to prevent sleep, and to wear upon the patient, and perhaps to cause vomiting, something must be done towards affording relief. If the cough is very severe indeed, nothing will control it except preparations of opium; but in the use of opium, get along with as little as possible. When less severe, sometimes hydrocyanic acid will check the cough; and there are several other remedies that may be employed for the same purpose. In some cases, sanguinaria is a remedy of considerable value. It is hard to be certain as to the exact effect of this remedy; but it has appeared to me that in a case in which the bronchitis predominates over the pneumonia, the sanguinaria is a remedy of considerable value in checking the cough.

The safest method of administration is to begin with five or ten drop doses of the tincture, and gradually increase to twenty or thirty drops every three or four hours. Vomiting may be produced if large doses are used at the outset.

In such cases as this, one of the best things that can be done is to remove the patient, if possible, to a better climate. For, there is here a reasonable chance for recovery, and a change of climate would be beneficial.

In those cases where the disease is very far advanced, and one or both lungs involved to a considerable extent, change of climate is hardly to be thought of.—*The Medical Record.*

